# Fluorescent carbon dots driven from ayurvedic medicinal plants for cancer cell imaging and phototherapy

**DOI:** 10.1016/j.heliyon.2019.e02483

**Published:** 2019-09-30

**Authors:** Ramhari Meena, Ranvir Singh, Gobinath Marappan, Garima Kushwaha, Narendra Gupta, Rekhraj Meena, Jay Prakash Gupta, Raja Ram Agarwal, Nighat Fahmi, Omkar Singh Kushwaha

**Affiliations:** a Department of Chemistry, University of Rajasthan, Jaipur, Rajasthan, 302004, India; b Nanoscience and Technology, Bharathiar University, Coimbatore, 641046, India; c Department of Biotechnology, Indian Institute of Technology, Roorkee, 247667, India; d Trident Diagnostics Center, Trivenee, Jaipur, 302015, Rajasthan, India; e Department of Radiology, Fortis Escorts Hospital, Jaipur, 302017, Rajasthan, India; f Madan Mohan Malviya Government Ayurvedic College and Hospital, Udaipur, Rajasthan, 313001, India; g Dr. Sarvepalli Radhakrishnan Rajasthan Ayurved University, Jodhpur, 342037, Rajasthan, India; h Department of Chemical Engineering, Indian Institute of Technology Madras, Chennai, 600036, India

**Keywords:** Biomedical engineering, Cancer research, Chemical engineering, Materials chemistry, Materials science, Medical imaging, Nanotechnology, Plant biology, Nanomaterials, Materials application, Materials characterization, Materials processing, Materials synthesis, Surface chemistry, Ayurveda, Cancer, Medicinal plants, Imaging, Therapy, Nano-dots

## Abstract

Ayurveda based nanomaterials are recently conceptualized phenomena for biomedical applications especially for imaging and treatment of in vitro cancer cell. Wide range florescent (blue to red emission) quantum dots are versatile materials for imaging and sensing applications. Various procedures and precursors of fluorescent carbon quantum dots (CQDs) are well established and documented in the literature. However, expensive precursors and production, and time consuming process limit their economical design that need to be addressed. Herein, we report a cost effective simple route for fluorescent CQDs by using affordable ayurvedic plant's precursors such as Azadirachta Indica, OcimumTenuiflorum and Tridax Procumbens. Obtained quantum dots from ayurvedic plant leaves namely CQDs-1 (AzadirachtaIndica), CQDs-2 (OcimumTenuiflorum) and CQDs-3 (TridaxProcumbens) showed homogeneous size distribution (∼6–12 nm) and green fluorescent nature, average photo-stability, biocompatibility (more than 85 %), cancer cell imaging and promising phototherapy for cancer and bacterial cell lines.

## 1 Introduction

In today's scenario, natural sources like ayurvedic plant leaves are highly attractive for producing various materials for agriculture and biomedical applications due to their biocompatibility and known natural ability [1, 2, 3]. Specially, fabricating carbon dots from ayurvedic plant leaves is a new and upcoming development having huge potential in the field of biomedical diagnosis [4, 5, 6]. However, the biocompatibility and multifunctionality of carbon quantum dots (also known as carbon nano-dots) are unresolved question so far in diagnosis and therapeutic applications [7, 8, 9, 10]. Previously, carbon derived nanomaterials namely graphene oxide sheets, graphene, carbon quantum dots, carbon fibers have been recognized as promising platforms for biomedical and electrochemical applications, each having its own advantages and challenges from synthesis to applications [11, 12, 13, 14, 15, 16, 17, 18, 19, 20]. In addition to biomedical applications, sensing, imaging and ion detection of fluorescent quantum dots are deeply understood and observed as attractive probes for diagnostics applications with highly reproducible outcomes [21, 22, 23, 24, 25]. As far as imaging is concerned, the high optical property of these carbon nanostructures attract the researchers in medical science [26, 27, 28]. So far, “Top-down” and “Bottom-up” approaches are well known to design fluorescent graphene/carbon quantum dots (GQDs/CQDs) from various commonly recognized carbon sources viz., sugarcane, plant leaf, bread, jaggery, corn flakes or biscuits, food caramels, etc. [29, 30, 31, 32, 33, 34, 35, 36, 37] Moreover, absorbance in broad near-infrared range and emission property of carbon quantum dots makes them attractive and suitable for cell imaging and phototherapy [38, 39, 40]. However, the known reported methods appear to be little too expensive and time consuming that produce the relatively low to high product yield of these dots. Additionally, controlling the optical property and biocompatibility of carbon quantum dots without surface functionalization is a challenging task so far [41, 42, 43, 44, 45]. Thus, the simplified and economically sustainable synthesis of biocompatible carbon quantum dots became significant to further their utilization for biomedical applications.

Here in the present manuscript, we are reporting an reasonably cost-effective and considerably easy route to obtain fluorescent carbon quantum dots [20, 44] from ayurvedic medicinal plants leaves viz., CQDs-1 (obtained from Azadirachta Indica),CQDs-2 (obtained from Ocimum Tenuiflorum) and CQDs-3 (obtained from Tridax Procumbens). The obtained CQDs are characterized by using various physicochemical techniques. Prepared CQDs are observed to be green fluorescent in nature, photo-stable, decently water dispersible and biocompatible. Selected precursors for CQDs synthesis have significant impact (in Indian Ayurveda system of treatment of diseases) which is highly cost effective and easily available across the country. In addition to this, *in-vitro* cell imaging, light based phototherapy on cancer cells lines and antimicrobial examinations have also been demonstrated successfully and discussed. Hence, the multifunctional performances of single probe viz., CQDs make them attractive for ayurvedic/natural nanomedicine.

## 2 Experimental

All glassware are washed with aqua regia (HCl: HNO3 = 3:1) carefully and rinsed with double distilled water before using them for synthesis. Analytical grade of chemicals and Milli-Q grade water are used for all experiments. Sulphuric acid (98 % H_2_SO_4_), Sodium carbonate (95 % Na_2_CO_3_) and Sodium Hydroxide (99 % NaOH) are purchased from Fischer Scientific limited Mumbai, India. Dulbecco's modified Eagle's medium (DMEM), fetal bovine serum (FBS), phosphate-buffered saline (PBS), antibiotic-antimycotic solution were procured from HiMedia Laboratories Pvt. Ltd, India [20, 44]. The leaves of medicinal plants Tulsi (OcimumTenuiflorum), Akal Kohadi (TridaxProcumbens) and Neem (Azadirachta India) were collected from Jaipur, the capital of State Rajasthan, India.

The transmission electron microscope (TEM) measurements were done for size distribution that was operated at 200 keV. Ultraviolet-Visible (UV-Vis) spectroscopy measurements were performed on a Jasco UV-Vis-Near Infra-Red (UV-Vis-NIR; Model V570) dual beam spectrometer operated at a resolution of 2 nm. PL spectra were acquired using a Cary eclipse fluorescence spectrophotometer. Fourier transform infrared (FTIR) spectra for the powder sample were recorded by Perkin-Elmer Spectrum One instrument, operated in the diffuse reflectance mode, at a resolution of 2 cm^−1^. To obtain a good signal to noise ratio, 256 scans were taken in the range 1200–3000 cm^−1^. Fluorescence microscopy images were taken by using a Carl Zeiss inverted fluorescence microscope model AXIO OBSERVER.ZI at different filters.

### 2.1 Simple synthesis of carbon quantum dots (CQDs)

The emissive CQDs were obtained by following earlier described methods, with some modifications adopted from our previously reported literature [20, 44]. The CQDs were synthesized separately from each ayurvedic plant by using the protocols from the following method. 100–120 mg of carbon ash from natural sources (obtained at 900 °C from the fibrous cellulosic leaf powder of individual plant) was mixed with the total 25 mL of concentrated H_2_SO_4_ and HNO_3_ and the mixture was left for 2–4 h for sonication so that uniformity in the resultant mixture should be observed. Thereafter, the mixture was stirred for 1 day followed by addition of 0.5 L water and pH adjustment to 7–8 by the subsequent addition of the aqueous Na_2_CO_3_ and NaOH to diluted the reaction mixture. In order to remove the salts in the form of a precipitate, the reaction solution was then subjected to slow stirring in an ice bath for several hours (about 40 h).

### 2.2 Photostability and aqueous dispersibility test of obtained CQDs

To evaluate the photostability and dispersibility, 2 mL aqueous solutions of prepared quantum dots viz., CQDs-1, CQDs-2 and CQDs-3 (0.5 mg/mL concentration) were treated at various temperatures (37–70 °C), pH, and irradiated with 365 nm UV lamp at different time points (2–24 h). After each exposure, 1 mL of the respective solution was analyzed through photoluminance spectra. Luminance property of above mentioned dots were analyzed after 30 days in order to get estimation of the photostability of each system in addition to the relative stability. Additionally, the aqueous dispersibility of these CQD systems was examined in visible light through digital photographic analysis.

### 2.3 Photothermal transduction and *in vitro* cytotoxicity

The time dependent photothermal performance of CQDs-1, CQDs-2 and CQDs-3 was tested at (0.5 mg/mL). Aliquots (150 μL) were deposited into cell culture plates and then wells were exposed with 750 nm continuous wave NIR light (0.5 W) for 10 minutes. Cytotoxicity study of CQDs was carried out on NIH3T3 fibroblastic normal cells that were cultured (1 × 10^4^ cells per well) in DMEM media supplemented with 10% FBS, 1 % penicillin and 1 % streptomycin in 5 % CO_2_ atmosphere at 37 °C. After 24 h incubation, 100 μl of different concentration (5–100 μg/mL dispersed in media) of CQDs-1, CQDs-2 and CQDs-3 were added into wells. Following 24 h incubation, wells were washed off with PBS and 20 μl of MTT dye was added into the respective system. Formazan crystals formed after 4 h were dissolved by 200 μL of DMSO. Optical absorbance was recorded at 570 nm and 690 nm using microplate reader (Tecan Infinite 200 PRO). Percentage cell viability was calculated in reference to untreated cells (control).

### 2.4 *In vitro* photoluminance and cancer cell imaging

HeLa cancer cells (1 × 10^4^cells per 96 well) were cultured in Dulbecco's Modified Eagle's Medium (DMEM Gibco, Carlsbad, CA, USA) supplemented with 10 % Fetal Bovine serum and penicillin/streptomycin, under 5 % CO_2_ atmosphere at 37 °C. After 24 h incubation, these wells were washed off with PBS and 100 μl of CQDs-1, CQDs-2 and CQDs-3, (0.5 mg/mL) added and after 12 h of incubation the PL spectra were recorded to examine the emission property of treated dots. Further these wells were washed off with PBS to remove unbound particles and 4% paraformaldehyde solution was added to the cells that was further stained with 4,6-diamidino-2-phenylindole (DAPI). DAPI dye is known to precisely accumulate in the nuclei of cancer cells. A cover slip was then mounted over a drop of 70 % glycerol on the glass slide to fix the cancer cells. Images were captured using a fluorescence microscope (Axio Observer Z1, Carl Zeiss).

### 2.5 *In vitro* photothermal cancer and bacteria therapy

HeLa cancer cells (1 × 10^4^cells per 96 well) were cultured in Dulbecco's Modified Eagle's Medium (DMEM Gibco, Carlsbad, CA, USA) supplemented with 10 % Fetal Bovine serum and penicillin/streptomycin, under 5 % CO_2_ atmosphere at 37 °C. After 24 h incubation, these wells were washed off with PBS and 100 μl of CQDs-1, CQDs-2 and CQDs-3 (0.5 mg/mL) and after 12 h of incubation, these wells were washed off with PBS to remove unbound particles and exposed with 10 minutes of 750 nm NIR light (0.5 W).

To examine the NIR light exposure on bacteria, E. coli culture (NCIM 2931) was sourced from the National Collection of Industrial Microorganisms (NCIM), CSIR-National Chemical Laboratory, Pune, India and was stored as agar slant at 4 °C (not exceeding 2 weeks). For every experiment, a loop-full of bacterial culture was taken and inoculated in fresh LB medium and grown at standard culture conditions of 37 °C at 180 rpm shaking speed. The overnight grown culture of E. coli cells was added into fresh LB medium. After the growth, 100 μl of CQDs-1, CQDs-2 and CQDs-3 (0.5 mg mL^−1^) were added and exposed with 10 minutes of 750 nm NIR light (0.5 W), further the plates were incubated additionally for for 8 h at 37 °C to screen for bacterial cell growth inhibition.

## 3 Results and discussion

**Fig. 1:**
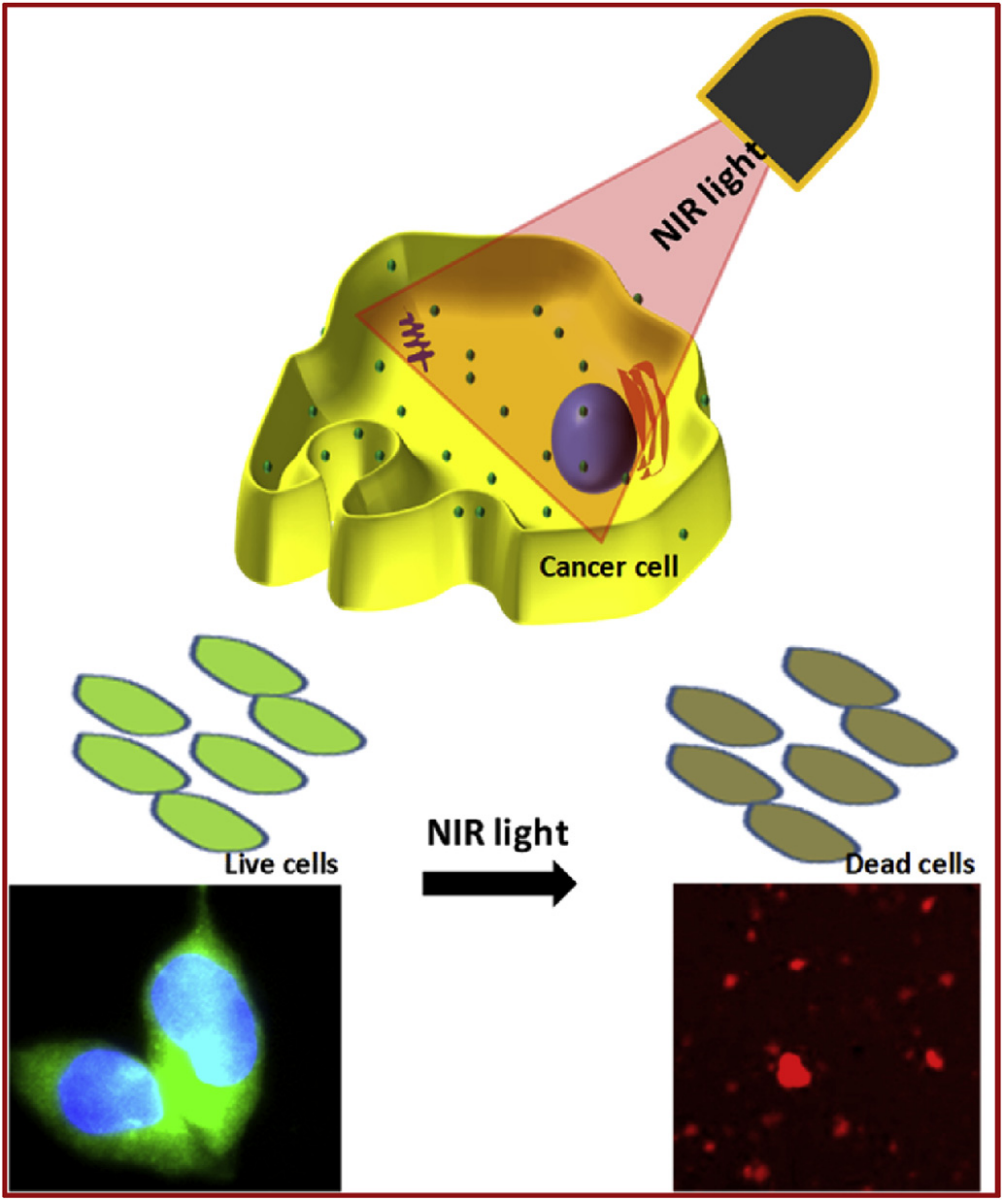
Green fluorescent carbon quantum dots (CQDs) for cell labeling and Near Infra-Red (NIR) light exposed therapy.

**Fig. 2:**
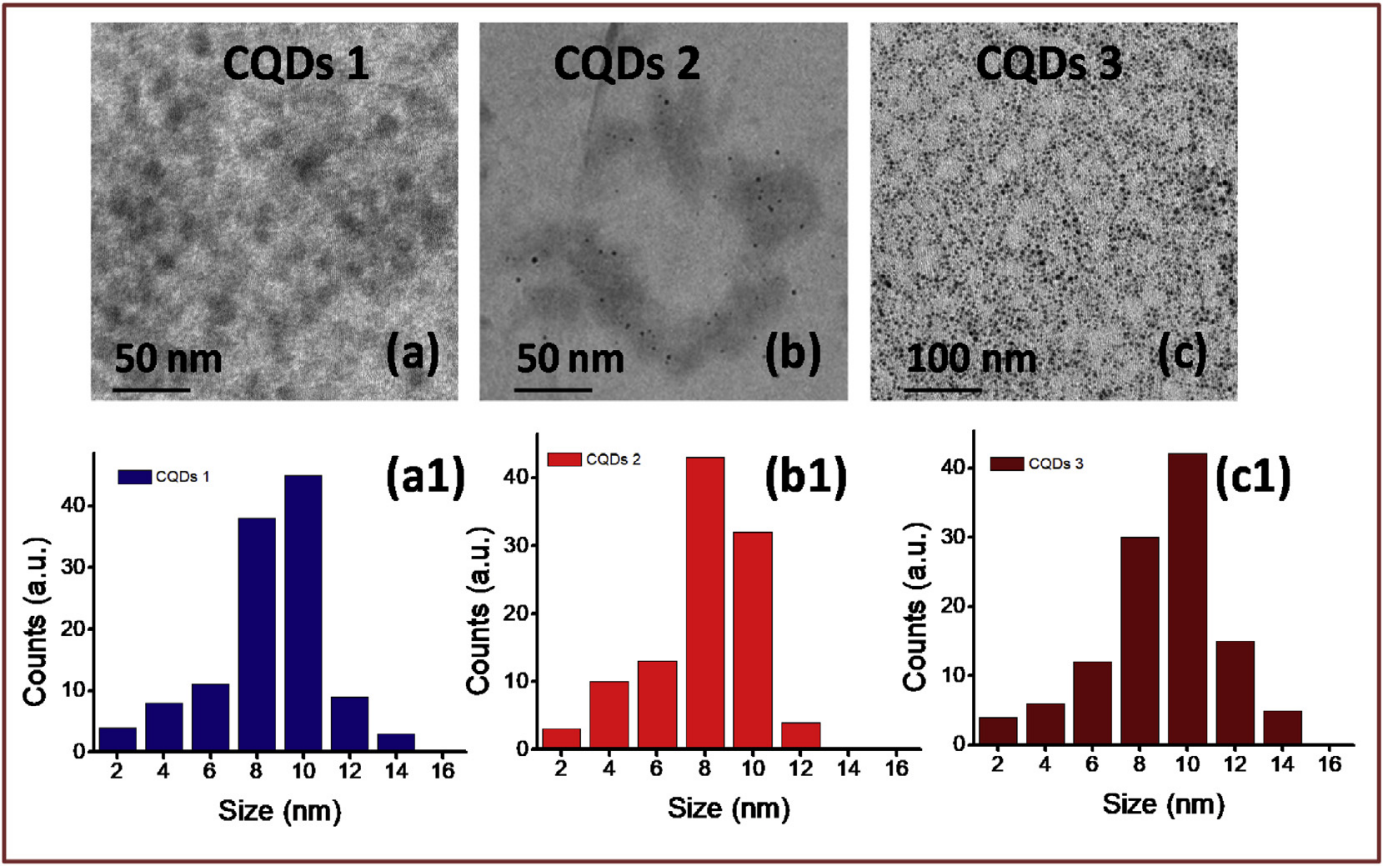
(a–c) TEM images and (a1–c1) particle size histogram of obtained fluorescent carbon quantum dots namely CQDs-1 (AzadirachtaIndica), CQDs-2 (OcimumTenuiflorum) and CQDs-3 (TridaxProcumbens) calculated from TEM images.

**Fig. 3:**
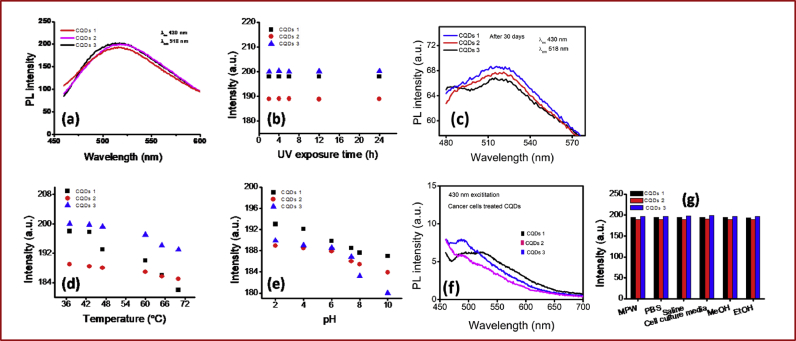
(a) Photoluminance (PL) spectra at room temperature on first day of CQDs preparation under 430 nm excitation, (b) time dependent PL emission intensity under 365 nm UV light exposure, (c) photoluminance (PL) spectra at room temperature on 30^th^ day of CQDs preparation under 430 nm excitation, (d) temperature dependent (37–70 °C) PL emission intensity, (e) pH dependent (2–10) PL emission intensity, (f) PL spectra at room temperature of cancer cell treated CQDs under 430 nm excitation and (g) PL emission intensity in various media/solvents of obtained quantum dots namely CQDs-1 (AzadirachtaIndica),CQDs-2 (OcimumTenuiflorum) and CQDs-3 (TridaxProcumbens).

Fluorescent carbon dots were obtained from natural sources viz., CQDs-1 (Azadirachta Indica), CQDs-2 (Ocimum Tenuiflorum) and CQDs-3 (Tridax Procumbens) through simple design for cell labeling and near infrared (NIR) light mediated therapy as shown in Fig. 1. The synthesis procedure of these dots was adopted from previously reported works [20, 44]. Prepared CQDs-1 to CQDs-3 show a size of about 6–10 nm with mean size of ∼10 nm and amorphous nature (TEM images and histogram profiles in Fig. 2 a–c and Fig. 2 a1-c1 and see ESI Fig. S1). Spectroscopic measurements viz., absorbance and fluorescence spectra have been demonstrated to understand the optical properties of these carbon quantum dots (CQDs namely in the manuscript as CQDs-1, CQDs-2 and CQDs-3) that confirmed the photostability and light to heat generation ability. Fluorescent/emissive nature of all three obtained CQDs was confirmed through photoluminescence (PL) spectra showing emission ∼518 nm with 430 nm excitation wavelength as shown in Fig. 3 a. Captured digital photographs of CQDs at various time points stipulate their fluorescent property and good dispersion ability in aqueous media as given in Fig. S2. The obtained CQDs were treated with UV light to examine the emission stability as shown in Fig. 3 b. 365 nm of UV light unable to change the fluorescent property of synthesized green emissive CQDs due to localized electronic transition in quantum dots framework. Further, the fluorescent property of designed CQDs was tested after 30 days that showed some observable changes in the fluorescence intensity as shown in Fig. 3c, revealed the moderate to high photostability of quantum dots obtained from various sources. The fluorescent stability was further supported with PL of these CQDs treated at various temperatures (37–70 °C, see Fig. 3 d). pH dependent (pH 2–10) photoluminance of obtained CDQs was analyzed as shown in Fig. 3 e demonstrating the higher intensity in cancer cell environment which reduced at higher pH due to surface passivation and quenching by high potential of hydroxyl anions in aqueous media.

Fig. 3 f shows the PL spectra of CQDs treated cancer cells lines with poor fluorescence intensity indicating the cellular binding or labeling supported with fluorescence microscopic images as explained here. These QDs, namely CQDs-1 (Azadirachta Indica), CQDs-2 (Ocimum Tenuiflorum) and CQDs-3 (Tridax Procumbens) were dispersed in various media or solvents viz., Millipore water (MPW), Phosphate Buffer Solution (PBS), Saline, cell culture media, MeOH and EtOH which showed good PL intensity and dispersion of CQDs as shown in Fig. 3 g. From the dispersion examination, we could not observe an obvious precipitation of CQDs solutions, particle aggregation and particle settling of CQDs solutions that indicate nice dispersion ability in aqueous media due to their hydrophilic functional groups and good surface chemistry (see Fig. S2). UV-Vis absorbance spectra of obtained CQDs from medicinal plants showed two UV absorption peaks that were noticed at λ_max_ 218 nm and λ_max_ 315 nm due to the π-π٭ transition of the C=C band and n-π٭ transition of C=O band respectively as shown in Fig. S3. The presence of functional group was validated by the FTIR analysis, the spectra is shown in Fig. S4. The O–H stretching vibrations (3324-36141 cm^−1^), –OH stretching peaks between 900-1026 cm^−1^ confirmed the presence of hydroxyl functional groups. Vibrations between 1500-1600 cm^−1^ are assigned to C=O stretching and N–H bending of CONH group. 2819 cm^−1^ and 2889 cm^−1^peaks attributed the -C-H stretching vibrations.

**Fig. 4:**
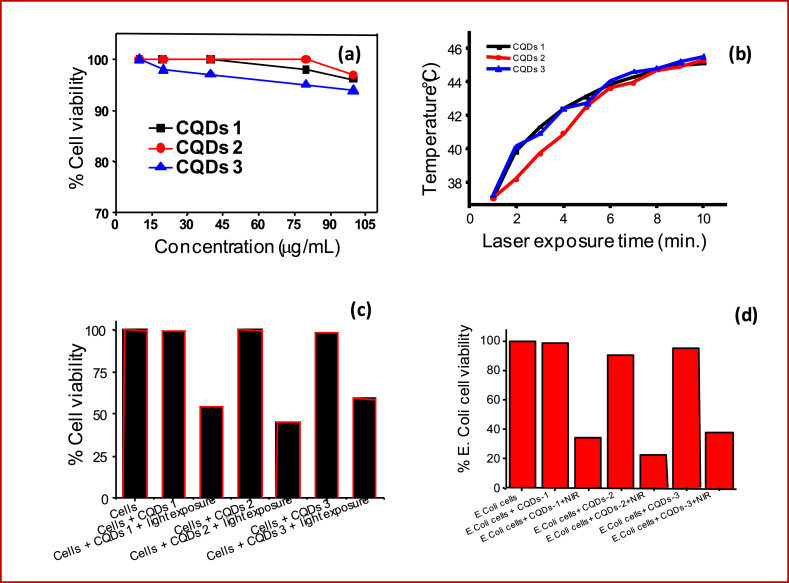
(a) % Cell viability with normal cell lines viz., NIH3T3, (b) time dependent NIR light responsive photothermal response, (c) NIR light tested *in vitro* photothermal cancer therapy and (d) NIR light mediated *in vitro* photothermal bacteria therapy using emissive CQDs-1, CQDs-2 and CQDs-3.

Prior to cell imaging, phototherapy and antimicrobial study of fabricated CQDs, the biocompatibility MTT test was carried out on NIH-3T3 normal cells. MTT assay has been adopted from previous report with some modification [20, 44] that is done on 10,000 count per well of cells in 96 well plate at various concentration of obtained CQDs (10–100 μg/mL, Fig. 4 a). After 24 h of incubation, 100 μL of CQDs from each set of concentration was added into cell contained 96 well plates. CQDs treated normal cells were further incubated for 24 h without changing the culture media during the incubation. Following observations were made for cell viability analysis which was determined by the addition of MTT (10 μL). MTT mixed 96 plates was incubated for an additional 4 h at 37 °C and 5 % CO_2_. Blue solution of cells converted into pink indicating the viability of normal cell lines. 100 μl of DMSO was added into each well to dissolve the Formazan crystals and the absorbance was recorded to confirm the cell cytotoxicity. About 80–85 % cell viability has been calculated for designed CQDs. The viability test could conclude that the prepared CQDs have no significant toxicity on the normal NIH-3T3 cells.

Next, light to heat generation photothermal transduction experiment of designed CQDs was demonstrated at 0.5 mg/mL (100μL) concentration using 750 nm of light exposure with 0.5 W power for the 10 minutes as shown in Fig. 4 b. The temperature variation was recorded at various time points that revealed the thermal response of the obtained CQDs. Hyperthermia temperature (43 °C) was recorded in 6 minutes that further rose up to 46 °C in 10 minutes of light irradiation. This revealed that the obtained CQDs may be suitable for light mediated cancer and bacteria phototherapy that has been tested on HeLa cancer cell lines and E. coli bacteria as shown in Fig. 4 c and d. The light exposure on cancer cells before CQDs treatment showed good cell viability (98 %). Before light irradiation, about 98–100 % cell viability was observed in the case of CQDs treated HeLa cells whereas ∼45–59 % cell viability has been calculated after 750 nm light exposure due to the thermal effect of prepared CQDs. Similarly, in case of bacteria phototherapy, about 95 % cell viability was noticed without light exposure whereas about 80 % cell death has been calculated after light treatment (see Fig. 4 d and more details are given in supporting information section 1 and Figs. S5 and S6). The above observations demonstrated the significant impact of light mediated therapy on cancer and bacterial cells for better health care. Hence, a single platform has been used to understand the multifunctional ability of designed fluorescent CQDs for cancer and bacteria nanomedicine that can attract the huge audience of biomedical field and various medical researchers.

**Fig. 5:**
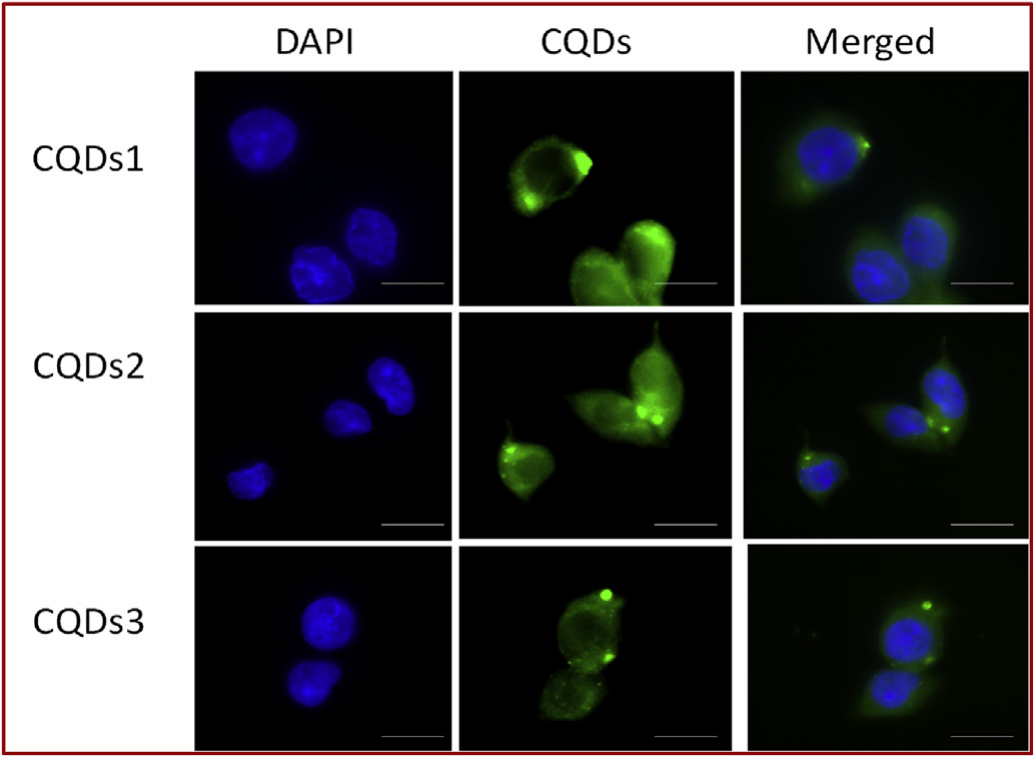
Cellular uptakes of fluorescent CQDs (CQDs-1 to CQDs-3) with HeLa cancer cell lines. Images are captured after 12 h of incubation.

HeLa cancer cells were selected for cell imaging measurements using fluorescent CQDs. 100μL aqueous solution of obtained fluorescent CQDs (0.5 mg/mL) were incubated with cancer cell lines up to 12 h to analyze the cellular uptake and intra cellular interlization of treated green emissive CQDs. Good aqueous dispersibility and quantum size of CQDs enhanced their cellular uptake ability. Fig. 5 shows the fluorescence microscopic images of CQDs treated HeLa cancer cells. The significant green fluorescence was observed in cell interior with good distribution indicating the intracellular localization of green fluorescent CQDs.

## 4 Conclusions

In summary, one pot, economical and ambient route of fluorescent CQDs preparation has been demonstrated. Nontoxic and emissive quantum dots were obtained from Indian Ayurvedic plant leaves being produced at ambient conditions. The obtained CQDs ensured (1) green fluorescence, (2) photo stability (3) water solubility and (4) biocompatibility. The designed CQDs also showed significant phototherapy on cancer and bacterial cells. The cost effective and easy availability of precursors of fluorescent CQDs make them attractive for imaging and therapeutics.

## Declarations

### Author contribution statement

Omkar Singh Kushwaha: Conceived and designed the experiments; Analyzed and interpreted the data; Contributed reagents, materials, analysis tools or data; Wrote the paper.

Ramhari Meena & Nighat Fahmi: Conceived and designed the experiments; Wrote the paper.

Gobinath Marappan, Ranvir Singh & Garima Kushwaha: Performed the experiments.

Narendra Gupta, Raja Ram Agarwal, Rekhraj Meena: Analyzed and interpreted the data; Contributed reagents, materials, analysis tools or data.

### Funding statement

Omkar Singh Kushwaha was supported by CSIR for research Grant 31/11(954)/2017-EMRI.

### Competing interest statement

The authors declare no conflict of interest.

### Additional information

No additional information is available for this paper.

## Appendix A Supplementary data

Nano Ayurveda_Supporting Information revised 2 Nano Ayurveda_Supporting Information revised 2
