# Supplementary material for: Fluorescent carbon dots driven from ayurvedic medicinal plants for cancer cell imaging and phototherapy
Source: Heliyon. 2019 Sep 30;5(9):e02483. doi: 10.1016/j.heliyon.2019.e02483 (PMC6819859; doi:10.1016/j.heliyon.2019.e02483)
Supplement: Nano Ayurveda_Supporting Information revised 2 [file mmc1.docx]

**Supporting Information**

**Fluorescent carbon dots driven from ayurvedic medicinal plants for cancer cell imaging and phototherapy**

Ramhari Meena^1^, Ranvir Singh^1^, Gobinath Marappan^2^, Garima Kushwaha^3^, Narendra Gupta^4^, Rekhraj Meena^5^, Jay Prakash Gupta^6^, Raja Ram Agarwal^6^, Nighat Fahmi^1^, Omkar Singh Kushwaha^7*^

^1^Department of Chemistry, University of Rajasthan, Jaipur, Rajasthan-302004, India

^2^Nanoscience and Technology, Bharathiar University, Coimbatore-641046, India

^3^ Department of Biotechnology, Indian Institute of Technology, Roorkee -247667, India

^4^ (i) Trident Diagnostics Center, Trivenee, Jaipur-302015, Rajasthan, India & (ii) Department of Radiology, Fortis Escorts Hospital, Jaipur-302017, Rajasthan, India

^5^Madan Mohan Malviya Government Ayurvedic College and Hospital, Udaipur, Rajasthan-313001, India

^6^Dr. Sarvepalli Radhakrishnan Rajasthan Ayurved University, Jodhpur-342037, Rajasthan, India

^7^ Department of Chemical Engineering, Indian Institute of Technology Madras, Chennai-600036, India

*Corresponding author

E-mail: [kushwaha.iitmadras@gmail.com](mailto:kushwaha.iitmadras@gmail.com); [oskushwaha.csirncl@gmail.com](mailto:oskushwaha.csirncl@gmail.com)

**Antibacterial study**

E. coli culture (NCIM 2931) was sourced from University of Rajasthan, India and was stored as agar slant at 4 °C (not exceeding 2 weeks). For every experiment, a loop-full of bacterial culture was taken and inoculated in fresh LB medium and grown at standard culture conditions of 37 °C at 180 rpm shaking speed.

The overnight grown culture of *E. coli* cells was added into fresh LB medium to give a concentration of O.D._600_ ca. 0.1. This culture was equally distributed in five different culture tubes. Three culture tubes were added with the three different as-synthesized carbon dots (R/ T/ N). The culture tubes without carbon dots and without inoculum were used as positive and negative control, respectively. The cultures were then grown at 37 ^o^C, 180 rpm for 8 h and the optical density at 600 nm was measured. The experiment was done in triplicates.

**Agar well diffusion method**

The LB agar media was autoclaved at 121 °C for 15 min and poured into sterile petri dish. Wells (0.5 cm) was made in all petri plates. The overnight grown culture of *E. coli* cells were used as seed culture. This seed culture was added into fresh LB medium to give a concentration of O.D._600_ ca. 0.1. 100 µl aliquots were taken out and spreaded on the petri plate. About 50 μL of the each of the three CQDs were added into the wells of different petri plates. The plates were incubated at 37 °C for 8 h to screen for bacterial cell growth inhibition. The diameter of the inhibition zone (cm) was measured.

**ROS determination**

**The *E. coli* suspension with O.D._600_ ca. 0.1 was treated as-synthesized carbon dots for 6 h. After incubation period of 6 h, the cells were pelletized by centrifugation at 10,000 g for 10 min at 4 °C, washed twice with PBS buffer and re-suspended in PBS buffer. DCF dye was added to above bacterial suspension at a final concentration of 5 µM and incubated for 1 h, at mild shaking. DCF dye is a non-fluorescent dye and gets converted to fluorescent dye in the presence of ROS; it determines the presence of intracellular ROS. The presence of fluorescent DCF dye was measured at an emission wavelength of 525 nm upon exciting the sample at an excitation wavelength 488 nm. The ROS concentration is considered directly proportional to the fluorescence intensity of the DCF dye.** The experiment was performed in triplicates.

**Supporting Figures with captions**

**Figure S1.** Selected area electron diffraction images of prepared CQDs.

**Figure S2.** Digital photographs of CQDs in day and UV (365 nm) light and also in various solvents/media showing their good photoluminance and dispersibility.

**Figure S3.** UV-Vis absorbance spectra of fluorescent CQDs.

**Figure S4.** FTIR spectra of fluorescent CQDs namely CQDs-1 (Azadirachta Indica, Neem), CQDs-2 (Ocimum Tenuiflorum, Tulasi) and CQDs-3 (Tridax Procumbens, Rookudi).

**Figure S5.** Qualitative and quantitative bacteria therapy analysis of fluorescent CQDs namely CQDs-1 (Azadirachta Indica, Neem), CQDs-2 (Ocimum Tenuiflorum, Tulasi) and CQDs-3 (Tridax Procumbens, Rookudi).

**Figure S6.** Qualitative anti-bacterial activity of fluorescent CQDs namely CQDs-1 (Azadirachta Indica, Neem), CQDs-2 (Ocimum Tenuiflorum, Tulasi) and CQDs-3 (Tridax Procumbens, Rookudi).
